# Evaluating the A-Subunit of the Heat-Labile Toxin (LT) As an Immunogen and a Protective Antigen Against Enterotoxigenic *Escherichia coli* (ETEC)

**DOI:** 10.1371/journal.pone.0136302

**Published:** 2015-08-25

**Authors:** Elizabeth B. Norton, Luis M. Branco, John D. Clements

**Affiliations:** Department of Microbiology and Immunology, Tulane University School of Medicine, New Orleans, Louisiana, United States of America; New York State Dept. Health, UNITED STATES

## Abstract

Diarrheal illness contributes to malnutrition, stunted growth, impaired cognitive development, and high morbidity rates in children worldwide. Enterotoxigenic *Escherichia coli* (ETEC) is a major contributor to this diarrheal disease burden. ETEC cause disease in the small intestine by means of colonization factors and by production of a heat-labile enterotoxin (LT) and/or a small non-immunogenic heat-stable enterotoxin (ST). Overall, the majority of ETEC produce both ST and LT. LT induces secretion via an enzymatically active A-subunit (LT-A) and a pentameric, cell-binding B-subunit (LT-B). The importance of anti-LT antibodies has been demonstrated in multiple clinical and epidemiological studies, and a number of potential ETEC vaccine candidates have included LT-B as an important immunogen. However, there is limited information about the potential contribution of LT-A to development of protective immunity. In the current study, we evaluate the immune response against the A-subunit of LT as well as the A-subunit’s potential as a protective antigen when administered alone or in combination with the B-subunit of LT. We evaluated human sera from individuals challenged with a prototypic wild-type ETEC strain as well as sera from individuals living in an ETEC endemic area for the presence of anti-LT, anti-LT-A and anti-LT-B antibodies. In both cases, a significant number of individuals intentionally or endemically infected with ETEC developed antibodies against both LT subunits. In addition, animals immunized with the recombinant proteins developed robust antibody responses that were able to neutralize the enterotoxic and cytotoxic effects of native LT by blocking binding and entry into cells (anti-LT-B) or the intracellular enzymatic activity of the toxin (anti-LT-A). Moreover, antibodies to both LT subunits acted synergistically to neutralize the holotoxin when combined. Taken together, these data support the inclusion of both LT-A and LT-B in prospective vaccines against ETEC.

## Introduction

Enterotoxigenic *E*. *coli* (ETEC) are a significant cause of diarrheal disease and death, especially in children in developing countries. In 2010, annual mortality from illness due to enterotoxigenic ETEC was estimated at 157,000 deaths (9 percent of all deaths attributed to diarrhea) and approximately 1 percent of all deaths in children 28 days to 5 years of age [1, 2]. Within susceptible populations, ETEC promotes a cycle of severe diarrheal disease, intestinal barrier dysfunction, and malnutrition, which impedes healthy growth, cognitive function, and long-term survival [3, 4]. ETEC is also well recognized as a cause of diarrheal disease in otherwise healthy adults while traveling to ETEC endemic areas [5] or by ingestion of contaminated food [6, 7], with a growing recognition that these infections can lead to chronic intestinal dysbiosis and post-infectious irritable bowel syndrome [8–10].

ETEC cause disease in the small intestine by means of colonization factors (CFs) and by production of a heat-labile enterotoxin (LT) and/or a small non-immunogenic heat-stable enterotoxin (ST). Overall, the majority of ETEC produce both ST and LT [5, 11–14]. LT induces secretion via an enzymatically active A-subunit (LT-A) and a pentameric, cell-binding B-subunit (LT-B) [15]. The A-subunit is made up of two components, A1 and A2. The A1-component (21 kD), the enzymatically active portion of the toxin, is non-covalently linked to the B-pentamer via the A2-peptide (7 kD) [16–18]. Expression of LT also facilitates bacterial adherence to epithelial cells and intestinal colonization [19, 20]. In areas where ETEC is endemic, the risk of recurrent diarrheal episodes drops after five years of age, concurrent with development of anti-LT antibodies [11, 21, 22]. The importance of anti-LT antibodies in protection from ETEC diarrheal disease has been demonstrated with several ETEC challenge studies in human adults and in a field study monitoring infants naturally receiving breast milk containing anti-LT IgA [23, 24]. These reports have strongly suggested that an anti-LT response provides significant immunity from LT-mediated secretion and possibly ETEC colonization; thus, an LT-related antigen should be an important component of an effective ETEC vaccine.

The B-subunit of LT is often presumed to be the immunodominant component of the toxin and potential vaccines against ETEC frequently include LT-B as one of the vaccine antigens. Evidence for B-subunit immunodominance in LT and the closely related cholera enterotoxin (CT) comes from antibody analyses of patient sera or animal studies with CT that found greater neutralizing capacity of anti-B subunit antibodies than anti-A subunit antibodies [25–29]. These findings led to led to the longstanding belief that the B-subunit is the major protective antigen against LT [24, 30–32].

The A-subunit of LT, although critical for enterotoxicity, has not been extensively evaluated for immunogenicity and is often overlooked as a potential protective antigen against ETEC, although there have been some attempts to genetically detoxify the A-subunit for use as a toxoid [33]. However, LT-A is clearly antigenic and a number of reports described production of anti-A monoclonal antibodies from clinical ETEC isolates [34–36]. Moreover, a 1984 study on patients recovering from cholera or ETEC infection found predominantly serum anti-B antibodies in cholera patients but equivalent serum anti-A and anti-B antibodies in ETEC patients (though both analyses were performed by ELISA using CT subunits) [37]. Thus, the relative importance of the A-subunit of LT as a protective antigen against ETEC is not well understood. The purpose of this study was to evaluate the immune response against the A-subunit of LT as well as its potential as a protective antigen when administered alone or in combination with the B-subunit of LT. We achieved this aim through a combinatorial approach, evaluating human serum responses, animal vaccination models, and toxin neutralization studies.

## Materials and Methods

### Purification of toxins and subunits

LT, LT-B, the non-toxic LT mutant LT(R192G/L211A), or dmLT, were produced from recombinant clones expressing LT, LT-B or dmLT based on LT from the human ETEC isolate *E*. *coli* H10407 as previously described [16, 17, 38]. Briefly, organisms were cultured overnight in 10-liters of Casamino Acids Yeast Extract medium, the cells were lysed, the lysate clarified by centrifugation, and LT-related proteins purified by galactose affinity chromatography. Proteins were stored lyophilized and freshly resuspended prior to use. His-tagged LT-A and His-tagged LT-A1 were prepared from solubilized inclusion bodies by HPLC with a nickel-affinity column as previously described [18]. The composition and purity of each protein was confirmed by SDS-PAGE and Limulus Amebocyte Lysate assay (Lonza, Inc.). The endotoxin content of the final products was <1 EU/mg.

### Human Serum Samples

Human sera pooled 10 days after ETEC challenge was provided by David A. Sack (Johns Hopkins University). Irradiated human patient sera from Sierra Leone was provided by Robert F. Garry (Tulane University). The Sierra Leone samples were collected from suspected Lassa virus febrile patients or afebrile controls between December 2009 and April 2010 [39]. De-identified samples were irradiated and stored at -20°C. 37 samples were included in the final analysis from patients aged 7–74 years. Commercially available human serum was purchased as a control (Sigma).

### Mouse Immunizations and sample collection

For these studies, we employed both *in vitro* (ELISA, CaCo-2 cell) and *in vivo* (patent mouse) assays following immunization by two different mucosal routes (intranasal, sublingual). Groups of 5 BALB/c mice each were intranasally immunized three times at weekly intervals with 10 μl saline containing 5 μg LT-A, LT-A1, LT-B, LT-A+LT-B (both at 5 μg) or dmLT. Alternatively, groups of mice were anesthetized with ketamine/xylazine and immunized with a single sublingual dose containing 5 μg LT-A, LT-A1, LT-B, LT-A+LT-B or LT in 10 μl of saline. All mice were sacrificed 21 days after the primary immunization. Serum was collected by cardiac puncture. Fecal samples were processed in protease buffer for supernatant antibody analysis as previously described [17]. This study was carried out in strict accordance with the recommendations in the Guide for the Care and Use of Laboratory Animals of the National Institutes of Health. The protocol was approved by the Tulane University Institutional Animal Care and Use Committee (Assurance Number A4499-01).

### Detection of anti-LT or anti-subunit antibodies

Anti-LT, -A, or -B serum antibody ELISAs were performed on individual serum samples with wells coated with 0.1 μg of antigen and quantified with an external mouse IgG or IgA standard, as previously described [40, 41], or analyzed for serum endpoint titer using a cut-off value of two-fold the OD value of control serum. Human ELISAs were performed using goat anti-human IgG-AKP (Sigma).

Immunoblots were performed as previously described with gels loaded with 1 μg of unboiled or boiled LT in all lanes [18]. Following electrophoresis and transfer, blots were probed with human sera diluted 1:1000 in blocking buffer. Blots were developed using goat anti-human IgG-HRP (Invitrogen) and TrueBlue peroxidase substrate (KPL).

### Neutralization of epithelial cell cAMP intoxication

Caco-2 cells (purchased from ATCC) were treated for 3 h with 0.1 μg trypsin-activated LT before the levels of intracellular cAMP were assessed using a cAMP assay kit (R&D Systems) [17, 18]. For neutralization of LT-induced cAMP, Caco-2 cells were treated with 10 μl containing 0.1 μg trypsinized LT pre-incubated overnight at 4°C with 10 μg GM1 monosialoganglioside (Sigma) or dilutions of rabbit anti-LTA, anti-LTB, anti-LT or negative control sera.

### Neutralization of toxin-mediated intestinal fluid secretion

The patent (non-occluded gut) mouse assay was performed as previously described [18]. Mice were fasted overnight and then intragastrically fed 0.5 ml saline containing 25 μg LT pre-incubated for 30 min at room temperature with dilutions of sera (amounts corresponding to those used in cAMP assay appropriately scaled for the increased amount of LT). After 3 h, animals were sacrificed, and the entire intestine from duodenum to anus was carefully excised. Tissue and carcasses were separately weighed and individual gut/carcass mass ratios calculated.

### Neutralization of toxin GM1 binding

Prevention of LT binding to GM1 was detected by ELISA as previously described [18]. Briefly, wells were coated with 10 μg of GM1 prior to addition of 0.1 μg LT with or without a 1 h pre-incubation with 1 μl serum at 25°C. LT binding was detected using anti-LT mouse serum prepared in our laboratory and anti-mouse IgG-AKP (Sigma). Absorbance was determined spectrophotometrically at 405 nm.

### Neutralization of toxin epithelial cell entry and ADP-ribosylation

Confluent monolayers of Caco-2 cells in 24-well plates were treated for 3 h with 1 μg LT with or without pre-incubation with 10 μl antiserum at 4°C. Cells were immediately washed 3x, lysed, then analyzed by Western Blot analysis for ADP-ribosylated proteins using His-tagged macrodomain rAF1521 (a generous donation from Andreas Ladurner) followed by anti-His-horseradish peroxidase (HRP) antibodies (Qiagen) or LT B-subunit using rabbit anti-B serum prepared in our laboratory and anti-donkey-HRP (Santa Cruz).

### Data analysis

Statistical analysis was performed using Prism (GraphPad Software, Inc.) for one-way ANOVA with Tukey’s Multiple Comparison post-test or Spearman’s Rank Order Correlation Coefficient (r) analysis. Unless otherwise indicated, *P*-values were coded as follows: *<0.05, **<0.01, ***<0.001.

## Results

In past studies, we successfully cloned the genes for LT, LT-A and LT-B and purified individual proteins free of cross-contamination [16–18, 38]. To critically examine the properties of both the A- and B-subunits, we performed a variety of analyses, including ELISA, Western blot, immunoblot, and toxin neutralization assay. Combining these techniques allowed us to examine both conformational and linear epitopes and different source of antigens (recombinant subunits or boiled, SDS-denatured LT toxin).

### ETEC-challenged human serum pool contains antibodies to both A- and B-subunits of LT

**Fig 1:**
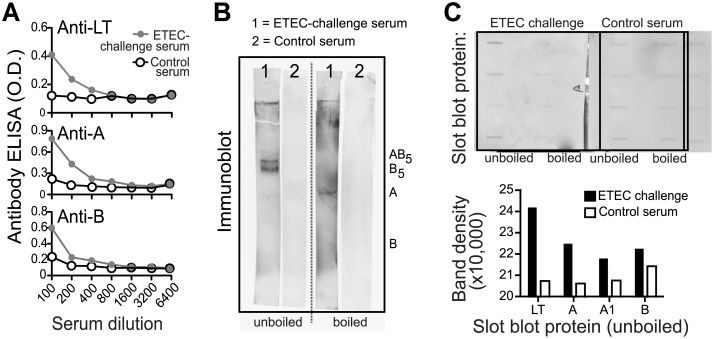
ETEC-challenged human serum pool contains antibodies to both A- and B-subunits of LT. (A) ETEC challenge serum (pooled 10 days after oral H10407 challenge) anti-LT, anti-A, and anti-B antibody responses detected by ELISA (gray line, circles) compared to commercially purchased control sera (black lines, open circles) using dilutions of each sample. (B) ETEC-challenge serum (1) or control serum (2) immunoblot testing for anti-LT antibodies using unboiled LT-loaded lanes or boiled LT-loaded lanes. In unboiled SDS-PAGE gels, LT runs as an 84 kD polymeric protein, pentameric B-subunit (56 kD), and LT-A (28 kD). When boiled and subjected to SDS-PAGE, LT separates into LT-A (28 kD) and monomeric LT-B (11.5 kD). (C) ETEC-challenge serum or control serum anti-LT, anti-A, or anti-B responses detected with a modified Immunoblot using a slot blot apparatus to load 0.1 μg protein (LT, A, A1, or B) with raw images (top) and quantified band density of these images for unboiled, loaded proteins graphed (bottom).

We first analyzed human immune serum for antibody response to LT holotoxin or subunits using a pool of sera from individuals challenged 10-days previously with ETEC isolate H10407 (O78:H11:K80 LT^+^ ST^+^), a prototypical strain of enterotoxigenic *E*. *coli* which reproducibly elicits diarrhea in human volunteer studies [42]. Commercially purchased human serum was included as a negative control. As seen in Fig 1, this ETEC-challenge serum tested positive for anti-LT, anti-A, and anti-B antibodies by ELISA (Fig 1A) in plates coated with native LT, LT-A or LT-B. The ELISA results indicated that antibodies to both the A-subunit and B-subunit were present in the sera of individuals infected with an LT-producing strain. The ELISA primarily detects antibodies against conformational epitopes and the use of A-subunit contaminated with traces of holotoxin or B-subunit could give a misleading impression of the presence of A-subunit antibodies. These studies used recombinantly produced LT-A and LT-B to reduce that possibility. In addition, we used immunoblots with boiled and unboiled preparations of LT to distinguish between the presence of antibodies against conformational and linear epitopes of LT in the H10407 challenge serum pool. In unboiled SDS-PAGE gels, LT runs as an 84 kD polymeric protein, pentameric B-subunit (56 kD), and LT-A (28 kD). When boiled and subjected to SDS-PAGE, LT separates into LT-A (28 kD) and monomeric LT-B (11.5 kD). When immunoblots with samples of unboiled LT were probed with H10407 challenge serum (Fig 1B), antibodies to LT, pentameric B (B5) and LT-A were observed, while predominantly anti-A antibodies were detected in the boiled LT preparation. This suggests that the anti-B response is against conformational epitopes, while the anti-A response is likely directed against both linear and conformational determinants. We confirmed this finding by probing immunoblots loaded with boiled or unboiled purified LT, LT-A, LT-A1, or LT-B with H10407 challenge serum (Fig 1C). Taken together, our results show that individuals infected with a wild-type ETEC strain developed antibodies to LT, LT-A, LT-A1 and LT-B.

### Serum samples from an ETEC-endemic area contain antibodies to both A- and B-subunits of LT

**Fig 2:**
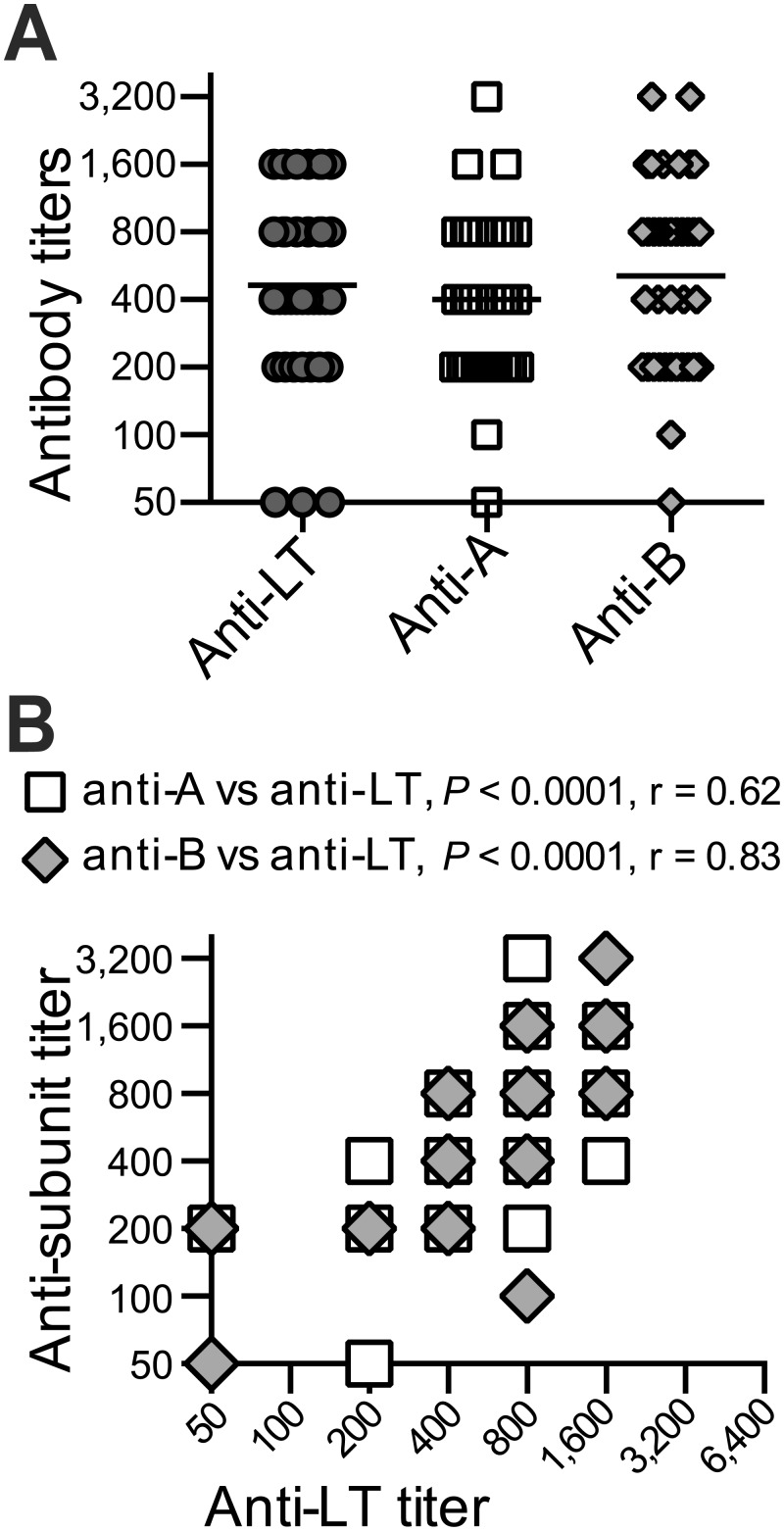
Serum samples from an ETEC-endemic area contain antibodies to both A- and B-subunits of LT. Human patient serum from Sierra Leone were evaluated for antibodies to LT, A-subunit, and B-subunit compared to commercially purchased control serum using endpoint titer analysis. (A) Anti-LT, anti-A, and anti-B ELISA antibody titers for all tested patient samples with geometric means indicated (lines). (B) Correlations between anti-LT and anti-A (open squares) or anti-B titers (gray diamonds) by Spearman’s Rank Order Correlation Coefficient (r) analysis. Antibody responses to LT holotoxin significantly correlated with both anti-A (*P*<0.0001) and anti-B (*P*<0.0001).

We next evaluated 37 human patient serum samples from Sierra Leone to evaluate antibodies in the setting of natural infection. While there is no epidemiological data specific to ETEC prevalence in this area, Sierra Leone is a developing country in sub-Saharan Africa, a region consistently associated within endemic ETEC [12, 24, 43]. In addition, current recommendations for travelers to Sierra Leone include precautions against contaminated food and water, identifying ETEC as the most common cause of travelers’ diarrhea [5]. These individual sera were collected from suspected Lassa virus febrile patients or afebrile controls, and while their ETEC exposure status is unknown, it is reasonable to assume that some or all of these individuals will have been infected with ETEC at some point. Individual serum samples were evaluated for the presence of antibodies to LT, LT-A and LT-B by ELISA. We first evaluated serial dilutions of individual patient serum in ELISA plates coated with LT, LT-A or LT-B to detect the presence of antibodies against the holotoxin and the individual subunits. The geometric mean titers of those sera were calculated and plotted by antigen (Fig 2A and S1 Fig) and approximately 70% of samples were positive for antibodies against one of the three antigens. Moreover, antibody responses to LT holotoxin significantly correlated with both anti-A (*P*<0.0001) and anti-B (*P*<0.0001) (Fig 2B), although there was heterogeneity within individual samples, including samples testing positive for anti-LT antibodies and both subunits or testing strongly for anti-LT antibodies and only anti-A- or anti-B antibodies. Similar to the H10407 challenge serum pool, antibodies to the B-subunit were predominantly conformational and were not apparent on immunoblots (using boiled LT), although bands corresponding to the A-subunit were readily detected (S1 Fig). Taken together, these results confirm our previous findings that individuals infected with ETEC developed antibodies to LT, LT-A, LT-A1 and LT-B.

### Both A- and B-subunits are immunogenic and induce anti-LT antibodies following mucosal immunization, with maximal responses observed when both subunits are present

**Fig 3:**
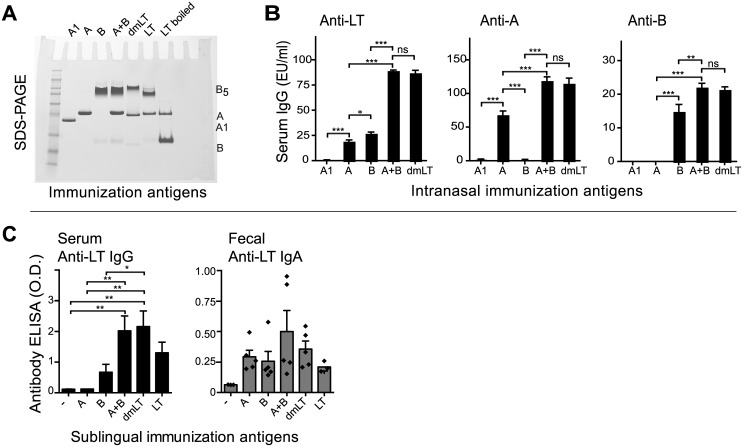
Both A- and B-subunits are immunogenic and induce anti-LT antibodies following mucosal immunization. For these studies, we employed an *in vitro* ELISA assay following immunization by two different mucosal routes (intranasal, sublingual). For intranasal immunizations, BALB/c mice were immunized weekly for three weeks with 10 μl saline containing 5 μg of protein antigens A1, A, B, A+B, or dmLT. For sublingual immunizations, BALB/c mice were sublingually immunized once with 10 μl saline containing 5 μg of protein antigens A, B, A+B, dmLT, or LT. All mice were sacrificed 21 days after the primary immunization. The integrity of the antigens was demonstrated by SDS-PAGE (A). Anti-LT serum IgG (B,C) or fecal IgA (C) responses were detected by ELISA. Significance testing for ELISA panels was done using one-way ANOVA with Tukey’s Multiple Comparison post-test. *P*-values were coded as follows: *<0.05, **<0.01, ***<0.001.

Analysis of sera from individuals experimentally or naturally infected with ETEC revealed that infection induces antibodies to both the A-subunit and B-subunit of LT. We next wanted to compare the antibody response after immunization with individual subunits alone or in combination. For these studies, we employed both *in vitro* (ELISA, CaCo-2 cell) and *in vivo* (patent mouse) assays following immunization by two different mucosal routes (intranasal, sublingual). For intranasal immunization, animals were immunized with LT-A, LT-A1, LT-B, LT-A and LT-B admixed together (LT-A+B), or the non-toxic A-subunit mutant dmLT. An SDS-PAGE gel of the purified proteins is shown in Fig 3A. Serum from these animals was collected and assayed in ELISA plates coated with native LT, LT-A or LT-B to examine the breadth of anti-LT responses induced by each immunogen. As seen in Fig 3B, LT-A1 was a poor immunogen and failed to induce antibodies by ELISA. By contrast, LT-A, which includes both A1 subunit and A2 domain, induced antibodies that recognized LT and LT-A. LT-B also induced antibodies to LT and to itself, but not LT-A. Surprisingly, the admix of LT-A and LT-B induced significantly higher responses against LT, LT-A and LT-B than either antigen alone, suggesting that the adjuvant function of LT-A was retained even when the molecules are dissociated. Specifically, LT-A is able to adjuvant the response to the B-subunit, even when the molecules are admixed and not present in the physiologically normal AB5 configuration. The anti-LT responses seen with the LT-A+B admix were equivalent to those seen with the non-toxic A-subunit mutant dmLT, which also retains immunogenic and adjuvant activity [17, 18]. Similar responses were observed when animals were immunized sublingually with LT-A, LT-B, the LT-A+B admix, or dmLT for both serum IgG and fecal IgA (Fig 3C). These results indicate that either the A- or B-subunit can be immunogenic when administered as a vaccine, but anti-toxin responses are maximized when both subunits are included in the immunization.

### Anti-A or anti-B subunit antibodies can neutralize LT toxicity, with maximal responses observed when antibodies against both subunits are present

**Fig 4:**
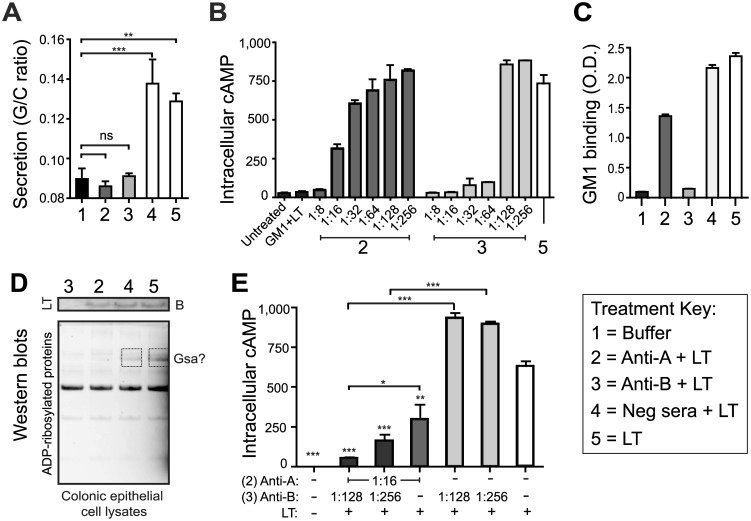
Anti-A or anti-B subunit antibodies neutralize LT toxicity, with maximal responses when both are present. (A) Neutralization of toxin-mediated intestinal fluid secretion in the patent mouse assay after intragastric feeding with buffer (1) or 25 μg LT combined with anti-A (2), anti-B (3), negative control sera (4), or toxin alone (5). (B,E) Neutralization of epithelial cell cAMP intoxication in Caco-2 cells after 3 h treatment with 0.1 μg LT pre-incubated with positive control GM1 monosialoganglioside (GM1+LT), various dilutions and combinations of anti-serum, or toxin alone. (C) Blocking of toxin GM1 binding by ELISA detection using 0.1 μg LT toxin pre-incubated with anti-sera or alone. (D) Western blot of Caco-2 cells lysates after 3 h treatment with 0.1 μg LT toxin pre-incubated with anti-sera. Blots were probed for ADP-ribosylated proteins using rAF1521 macrodomains (bottom blot) then re-probed for presence of the B-subunit using anti-B rabbit serum (top blot). Boxes indicate proteins with altered ADP-ribosylation, potentially Gsα. Significance testing for secretion and cAMP panels was done using one-way ANOVA with Tukey’s Multiple Comparison post-test. *P*-values were coded as follows: *<0.05, **<0.01, ***<0.001.

While LT-B facilitates cell binding and internalization of the toxin, LT-A mediates the intracellular enzymatic functions after proteolytic cleavage and disulfide bond reduction into the active A1 domain [15]. Within the cytoplasm of intestinal epithelial cells, the A1 domain ADP-ribosylates numerous cellular proteins, including Gsα, leading to irreversible activation of adenylate cyclase, cAMP accumulation, and deregulation of ion transport mechanisms that control luminal osmolarity and fluid secretion. Thus, there are a number of potential targets for protective antibodies within the steps leading to LT enterotoxicity. To determine how antibodies specific to each subunit might contribute to protection against LT toxicity, we employed two different assays—the patent mouse oral enterotoxicity assay and the CaCo-2 cell cAMP assay. For the patent mouse assay, animals were orally inoculated with an amount of native toxin sufficient to induce half-maximal fluid secretion after three hours (i.e., 25 μg LT). LT toxin was pre-incubated with rabbit anti-A serum, rabbit anti-B serum, negative control serum, or buffer. The rabbit polyclonal sera were produced in our laboratory against purified recombinant LT-A and LT-B. As seen in Fig 4A, both anti-A and anti-B were able to effectively neutralize the secretory activity of native LT in the patent mouse assay, indicating that both LT’s A-subunit and B-subunit are immunoprotective against LT-mediated diarrhea.

For the cAMP assay, CaCo-2 cells were treated with an amount of LT sufficient to cause maximal cAMP accumulation in 3 hours (i.e., 0.1 μg). For this assay, the LT was first pre-incubated with GM1 (positive control) or serial dilutions of rabbit anti-A or anti-B serum (Fig 4B). Interestingly, two different effects were observed. For anti-LT-A, neutralization of LT gradually diminished the further the serum was diluted, starting at 1:8 (100%) through 1:256 (0%). By contrast, anti-LT-B appeared to have a marked threshold where neutralization went from all to none in a single 2-fold dilution (64–128). This suggests that there is a threshold level of anti-B above which LT binding to the cell is blocked and any level below may not be effective.

We subsequently examined whether these antisera act to block binding or enzymatic activity of LT using ELISA plates coated with GM1 or an intracellular ADP-ribosylation assay. For both assays, LT was pre-incubated with rabbit anti-A, rabbit anti-B, negative control serum, or buffer prior to addition. Anti-B antibodies prevented GM1 binding (Fig 4C), entry of toxin into the cell (Fig 4D, top blot) and enzymatic activity (Fig 4D, lower blot). By contrast, anti-A antibodies only prevented the enzymatic activity of the toxin within epithelial cells. These results indicate antibodies to the B-subunit prevent toxin attachment and entry into intestinal cells, whereas antibodies to the A-subunit prevent enzymatic activity of the toxin within intracellular compartments.

We also examined potential synergy between anti-A and anti-B for toxin neutralization. For this assay, we combined anti-A and anti-B sera at dilutions that had not shown complete toxin neutralization in Fig 4B (e.g., 1:16 anti-A; 1:128–256 anti-B). As seen in Fig 4E, dilutions of anti-B at levels of 1:128–256 were unable to neutralize LT induction of cAMP, unless combined with anti-A. In addition, the neutralizing ability of anti-A was greatly enhanced by the presence of anti-B. Thus, anti-toxin responses are maximized when antibodies to both subunits are present.

## Discussion

The development of an effective vaccine against ETEC has been complicated by a number of factors, including the absence of a substantial developed country market to offset the cost of development, the focus on travellers as a primary vaccine target, the large number of potential serotypes and colonizing factor antigens, and the (relative) success of the whole cell B-subunit cholera vaccine. This first led to development of killed ETEC vaccine candidates combined with the B-subunit of cholera toxin as the primary anti-toxin component. More recent studies have utilized killed or live attenuated ETEC strains combined with the B-subunit of LT [44, 45]. There have been a few attempts to include an LT-A component, including an LT-based transcutaneous patch that successfully prevented LT-only ETEC diarrhea in travellers (although not all ETEC diarrhea) [40]. In addition, LT-A is difficult to purify free from contamination with LT-B and is insoluble in most aqueous buffers. As a result, most ETEC vaccine development has included the B-subunit as the sole anti-toxin component.

The purpose of this study was to evaluate the immune response against the A-subunit of LT as well as its potential as a protective antigen when administered alone or in combination with the B-subunit of LT. In order to avoid the issue of cross-contamination with trace amounts of competing proteins (often an issue in older studies using dissociation chromatography), we utilized recombinantly produced LT, LT-A and LT-B purified on pristine chromatography columns as antigens and immunogens in a series of *in vitro* and *in vivo* assays. We have extensive experience with purification and characterization of LT and its subunits as well as evaluation of their immunologic and physiologic properties [16–18, 31, 38, 46].

Our studies clearly show that both LT subunits are immunogenic. Human sera from individuals challenged with a prototypic wild-type ETEC strain as well as sera from individuals living in an ETEC endemic area had detectable antibodies against LT, LT-A, and LT-B detected by ELISA and Immunoblot (Figs 1 and 2 and S1 Fig). No clear immunodominant subunit for LT was identified, however, antibodies to the A-subunit often included both conformational and non-conformational epitopes, whereas those to the B-subunit were primarily conformational. This data clearly illustrates that LT’s A-subunit is naturally immunogenic like LT’s B-subunit, similar to past studies [34–37].

During ETEC bacterial infection, LT toxin expression occurs directly at gastrointestinal surfaces and possibly using specialized delivery systems such as outer membrane vesicles [47]. We have previously shown that the A-subunit of LT is also more susceptible than the B-subunit to proteolytic cleavage into an A1 domain and degradation [18]. Consequently, the immunogenicity of LT and its subunits could be distinct in the context of antigen vaccination as opposed to bacterial infection. In our murine immunization studies, we confirmed that robust antibody responses were detected after immunization with individual subunits, but these were maximized in combination with admixed A+B or AB5 constructs (Fig 3). A smaller serum response was seen with A-subunit immunization after sublingual immunization than occurred with intranasal immunization, as might be expected given the more complicated dynamics of antigen uptake at the sublingual epithelium (without the presence of a GM1-binding B-subunit) and presence of salivary proteases. The synergetic properties of admixed A+B with regards to higher levels of immunologic responses has been seen previously in our studies evaluating the adjuvant properties of A-subunit or admixed A+B [18]. This suggests that immunogenicity of LT toxin, much like adjuvanticity, is not dependent on the AB5 holotoxin structure, although induction of systemic antibody responses is maximal with at least two signals received from both A- and B-subunits.

A major conclusion of our study is that both subunits can be immunoprotective and neutralize LT-mediated diarrheal secretion (Fig 4). We also set up specific assays to evaluate at what point do antibodies neutralize or prevent the intoxication of epithelial cells, including GM1 binding, cellular entry, ADP-ribosylation, and cAMP accumulation (Fig 4). These assays represent a potential for improved evaluation of LT-neutralizing antibodies that is more quantifiable and specific than the historically used Y-1 adrenal cell assay assessing cellular rounding/non-rounding post-toxin treatment [33–36]. Polyclonal rabbit serum antibodies were able to neutralize the enterotoxic and cytotoxic effects of native LT by blocking binding and entry into cells (anti-LT-B) or the intracellular enzymatic activity of the toxin (anti-LT-A and anti-LT-B). Moreover, antibodies to both LT subunits acted synergistically to neutralize the holotoxin when combined. Taken together, these data support the inclusion of both LT-A and LT-B in prospective vaccines against ETEC.

A major question left unanswered by this study is which LT antigen form is optimal—an AB5 toxoid, like dmLT, or combinations of toxin-derived proteins, like LT-A+LT-B. Minimal differences were seen between dmLT and LT-A+LT-B in our mouse immunization studies at doses and routes evaluated. However, more extensive studies would be required to assess differences between these proteins in both formulation stability and vaccination outcomes when combined with other ETEC antigens. The question of LT antigen form becomes further complicated by delivery route and whether the adjuvant properties of an LT-derived protein [17, 18] are also desired. What is clear from these results is that antibody analyses that include LT holotoxin or both A- and B-subunits produces more informative results than that obtained with only the B-subunit. With these analysis techniques in mind, future ETEC vaccine studies can begin to answer these outstanding questions by utilizing combinations of LT antigen(s) with a specific immunization approach (e.g., oral live-attenuated or heat-killed vaccines in contrast to parenterally injected subunits or fusion proteins).

In conclusion, both A- and B-subunits contribute to immunoprotection against LT-mediated diarrhea, resulting in antibody responses that target unique aspects of toxin biology and provide enhanced toxin neutralization when combined. Therefore, inclusion of both subunit epitopes in ETEC vaccine candidates seems likely to promote a more robust neutralizing response to LT toxin than the B-subunit alone.

## Supporting Information

S1 Fig Serum samples from an ETEC-endemic area contain antibodies to both A- and B-subunits of LT. Human patient serum from Sierra Leone were evaluated for antibodies to LT, A-subunit, and B-subunit compared to commercially purchased control serum. (A) Human patient serum from Sierra Leone (black lines) or control serum (open circles) anti-LT, anti-A, and anti-B antibody responses detected by ELISA by 405 nm optical density (O.D.) (B) Human patient serum from Sierra Leone, control serum, or no serum immunoblot testing for non-conformational anti-LT antibodies to monomer A- or B-subunits. All lanes were loaded with boiled LT protein with location of A and B bands indicated.
(EPS)
