# Correction: Evaluating the A-Subunit of the Heat-Labile Toxin (LT) As an Immunogen and a Protective Antigen Against Enterotoxigenic *Escherichia coli* (ETEC)

**DOI:** 10.1371/journal.pone.0138938

**Published:** 2015-09-18

**Authors:** The PLOS ONE Staff

Fig 1 is incorrect. The authors have provided a corrected version here. The publisher apologizes for the error.

**Fig 1:**
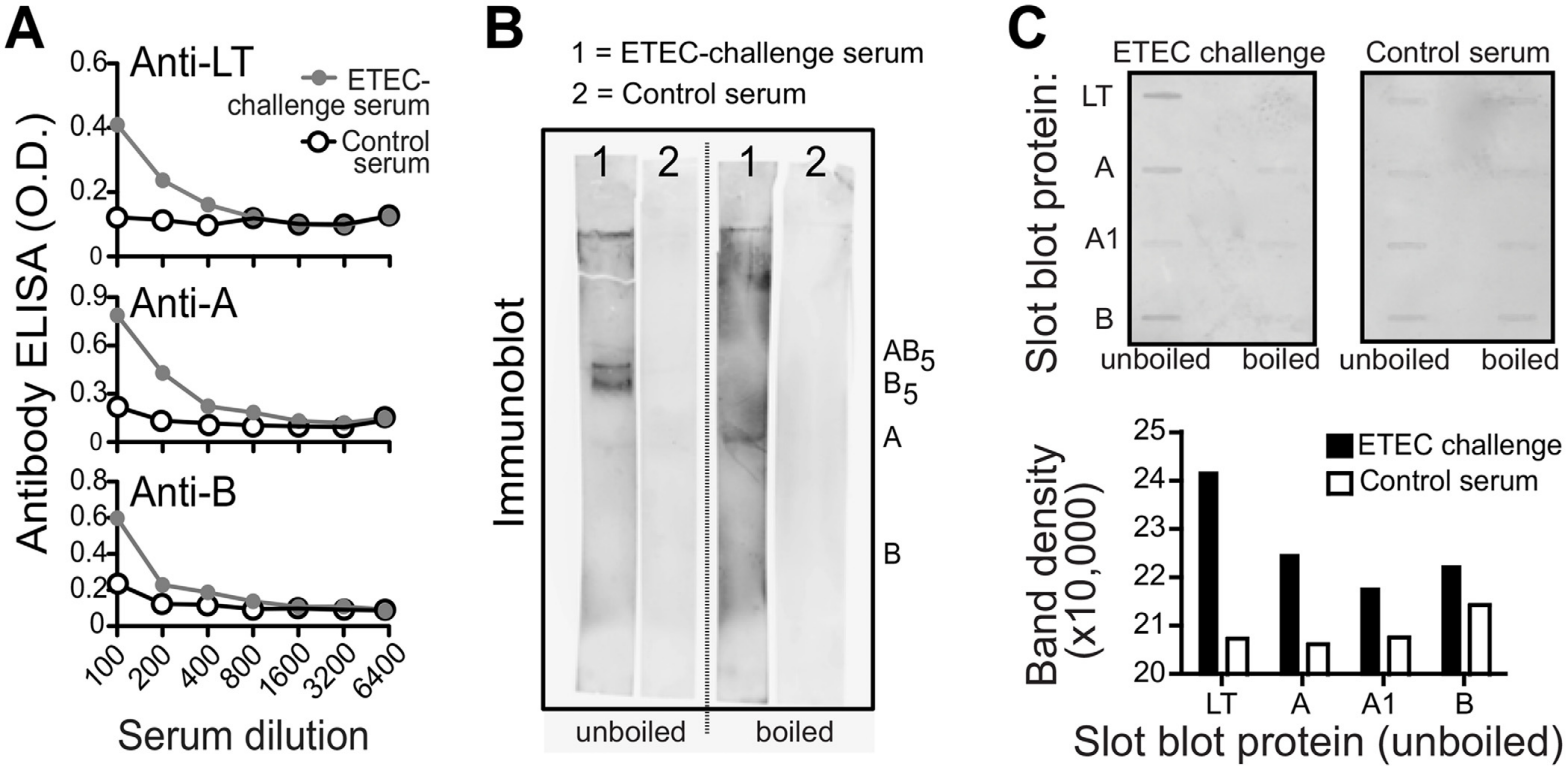
ETEC-challenged human serum pool contains antibodies to both A- and B-subunits of LT. (A) ETEC challenge serum (pooled 10 days after oral H10407 challenge) anti-LT, anti-A, and anti-B antibody responses detected by ELISA (gray line, circles) compared to commercially purchased control sera (black lines, open circles) using dilutions of each sample. (B) ETEC-challenge serum (1) or control serum (2) immunoblot testing for anti-LT antibodies using unboiled LT-loaded lanes or boiled LT-loaded lanes. In unboiled SDS-PAGE gels, LT runs as an 84 kD polymeric protein, pentameric B-subunit (56 kD), and LT-A (28 kD). When boiled and subjected to SDS-PAGE, LT separates into LT-A (28 kD) and monomeric LT-B (11.5 kD). (C) ETEC-challenge serum or control serum anti-LT, anti-A, or anti-B responses detected with a modified Immunoblot using a slot blot apparatus to load 0.1 μg protein (LT, A, A1, or B) with raw images (top) and quantified band density of these images for unboiled, loaded proteins graphed (bottom).
